# Supplementary material for: Smooth Interpolating Curves with Local Control and Monotone Alternating Curvature
Source: Comput Graph Forum. 2022 Oct 6;41(5):25–38. doi: 10.1111/cgf.14600 (PMC9827861; doi:10.1111/cgf.14600)
Supplement: Supplementary file 1 — Supplement Material [file CGF-41-25-s001.zip › Local-Smooth-Interpolating-MonoCurvature/extern/clothoids/submodules/Utils/ThirdParties/fmt/fmt-8.0.1.zip!/fmt-8.0.1/doc/html/syntax.html]

Format String Syntax — fmt 8.0.1 documentation


Toggle navigation

{fmt}

- 8.0.1
  - 8.0.1
  - 8.0.0
  - 7.1.3
- Contents
- Usage
- API
- Syntax
  (current)

# Format String Syntax¶

Formatting functions such as fmt::format() and
fmt::print() use the same format string syntax described in this
section.

Format strings contain “replacement fields” surrounded by curly braces `{}`.
Anything that is not contained in braces is considered literal text, which is
copied unchanged to the output. If you need to include a brace character in the
literal text, it can be escaped by doubling: `{{` and `}}`.

The grammar for a replacement field is as follows:

```
replacement_field ::=  "{" [arg_id] [":" (format_spec | chrono_format_spec)] "}"
arg_id            ::=  integer | identifier
integer           ::=  digit+
digit             ::=  "0"..."9"
identifier        ::=  id_start id_continue*
id_start          ::=  "a"..."z" | "A"..."Z" | "_"
id_continue       ::=  id_start | digit
```

In less formal terms, the replacement field can start with an *arg\_id*
that specifies the argument whose value is to be formatted and inserted into
the output instead of the replacement field.
The *arg\_id* is optionally followed by a *format\_spec*, which is preceded by a
colon `':'`. These specify a non-default format for the replacement value.

See also the Format Specification Mini-Language section.

If the numerical arg\_ids in a format string are 0, 1, 2, … in sequence,
they can all be omitted (not just some) and the numbers 0, 1, 2, … will be
automatically inserted in that order.

Named arguments can be referred to by their names or indices.

Some simple format string examples:

```
"First, thou shalt count to {0}" // References the first argument
"Bring me a {}"                  // Implicitly references the first argument
"From {} to {}"                  // Same as "From {0} to {1}"
```

The *format\_spec* field contains a specification of how the value should be
presented, including such details as field width, alignment, padding, decimal
precision and so on. Each value type can define its own “formatting
mini-language” or interpretation of the *format\_spec*.

Most built-in types support a common formatting mini-language, which is
described in the next section.

A *format\_spec* field can also include nested replacement fields in certain
positions within it. These nested replacement fields can contain only an
argument id; format specifications are not allowed. This allows the formatting
of a value to be dynamically specified.

See the Format Examples section for some examples.

## Format Specification Mini-Language¶

“Format specifications” are used within replacement fields contained within a
format string to define how individual values are presented (see
Format String Syntax). Each formattable type may define how the format
specification is to be interpreted.

Most built-in types implement the following options for format specifications,
although some of the formatting options are only supported by the numeric types.

The general form of a *standard format specifier* is:

```
format_spec ::=  [[fill]align][sign]["#"]["0"][width]["." precision]["L"][type]
fill        ::=  <a character other than '{' or '}'>
align       ::=  "<" | ">" | "^"
sign        ::=  "+" | "-" | " "
width       ::=  integer | "{" [arg_id] "}"
precision   ::=  integer | "{" [arg_id] "}"
type        ::=  "a" | "A" | "b" | "B" | "c" | "d" | "e" | "E" | "f" | "F" | "g" | "G" |
                 "o" | "p" | "s" | "x" | "X"
```

The *fill* character can be any Unicode code point other than `'{'` or
`'}'`. The presence of a fill character is signaled by the character following
it, which must be one of the alignment options. If the second character of
*format\_spec* is not a valid alignment option, then it is assumed that both the
fill character and the alignment option are absent.

The meaning of the various alignment options is as follows:

| Option | Meaning |
| --- | --- |
| `'<'` | Forces the field to be left-aligned within the available space (this is the default for most objects). |
| `'>'` | Forces the field to be right-aligned within the available space (this is the default for numbers). |
| `'^'` | Forces the field to be centered within the available space. |

Note that unless a minimum field width is defined, the field width will always
be the same size as the data to fill it, so that the alignment option has no
meaning in this case.

The *sign* option is only valid for number types, and can be one of the
following:

| Option | Meaning |
| --- | --- |
| `'+'` | indicates that a sign should be used for both positive as well as negative numbers. |
| `'-'` | indicates that a sign should be used only for negative numbers (this is the default behavior). |
| space | indicates that a leading space should be used on positive numbers, and a minus sign on negative numbers. |

The `'#'` option causes the “alternate form” to be used for the
conversion. The alternate form is defined differently for different
types. This option is only valid for integer and floating-point types.
For integers, when binary, octal, or hexadecimal output is used, this
option adds the prefix respective `"0b"` (`"0B"`), `"0"`, or
`"0x"` (`"0X"`) to the output value. Whether the prefix is
lower-case or upper-case is determined by the case of the type
specifier, for example, the prefix `"0x"` is used for the type `'x'`
and `"0X"` is used for `'X'`. For floating-point numbers the
alternate form causes the result of the conversion to always contain a
decimal-point character, even if no digits follow it. Normally, a
decimal-point character appears in the result of these conversions
only if a digit follows it. In addition, for `'g'` and `'G'`
conversions, trailing zeros are not removed from the result.

*width* is a decimal integer defining the minimum field width. If not
specified, then the field width will be determined by the content.

Preceding the *width* field by a zero (`'0'`) character enables sign-aware
zero-padding for numeric types. It forces the padding to be placed after the
sign or base (if any) but before the digits. This is used for printing fields in
the form ‘+000000120’. This option is only valid for numeric types and it has no
effect on formatting of infinity and NaN.

The *precision* is a decimal number indicating how many digits should be
displayed after the decimal point for a floating-point value formatted with
`'f'` and `'F'`, or before and after the decimal point for a floating-point
value formatted with `'g'` or `'G'`. For non-number types the field
indicates the maximum field size - in other words, how many characters will be
used from the field content. The *precision* is not allowed for integer,
character, Boolean, and pointer values.

The `'L'` option uses the current locale setting to insert the appropriate
number separator characters. This option is only valid for numeric types.

Finally, the *type* determines how the data should be presented.

The available string presentation types are:

| Type | Meaning |
| --- | --- |
| `'s'` | String format. This is the default type for strings and may be omitted. |
| none | The same as `'s'`. |

The available character presentation types are:

| Type | Meaning |
| --- | --- |
| `'c'` | Character format. This is the default type for characters and may be omitted. |
| none | The same as `'c'`. |

The available integer presentation types are:

| Type | Meaning |
| --- | --- |
| `'b'` | Binary format. Outputs the number in base 2. Using the `'#'` option with this type adds the prefix `"0b"` to the output value. |
| `'B'` | Binary format. Outputs the number in base 2. Using the `'#'` option with this type adds the prefix `"0B"` to the output value. |
| `'c'` | Character format. Outputs the number as a character. |
| `'d'` | Decimal integer. Outputs the number in base 10. |
| `'o'` | Octal format. Outputs the number in base 8. |
| `'x'` | Hex format. Outputs the number in base 16, using lower-case letters for the digits above 9. Using the `'#'` option with this type adds the prefix `"0x"` to the output value. |
| `'X'` | Hex format. Outputs the number in base 16, using upper-case letters for the digits above 9. Using the `'#'` option with this type adds the prefix `"0X"` to the output value. |
| none | The same as `'d'`. |

Integer presentation types can also be used with character and Boolean values.
Boolean values are formatted using textual representation, either `true` or
`false`, if the presentation type is not specified.

The available presentation types for floating-point values are:

| Type | Meaning |
| --- | --- |
| `'a'` | Hexadecimal floating point format. Prints the number in base 16 with prefix `"0x"` and lower-case letters for digits above 9. Uses `'p'` to indicate the exponent. |
| `'A'` | Same as `'a'` except it uses upper-case letters for the prefix, digits above 9 and to indicate the exponent. |
| `'e'` | Exponent notation. Prints the number in scientific notation using the letter ‘e’ to indicate the exponent. |
| `'E'` | Exponent notation. Same as `'e'` except it uses an upper-case `'E'` as the separator character. |
| `'f'` | Fixed point. Displays the number as a fixed-point number. |
| `'F'` | Fixed point. Same as `'f'`, but converts `nan` to `NAN` and `inf` to `INF`. |
| `'g'` | General format. For a given precision `p >= 1`, this rounds the number to `p` significant digits and then formats the result in either fixed-point format or in scientific notation, depending on its magnitude.  A precision of `0` is treated as equivalent to a precision of `1`. |
| `'G'` | General format. Same as `'g'` except switches to `'E'` if the number gets too large. The representations of infinity and NaN are uppercased, too. |
| none | Similar to `'g'`, except that the default precision is as high as needed to represent the particular value. |

The available presentation types for pointers are:

| Type | Meaning |
| --- | --- |
| `'p'` | Pointer format. This is the default type for pointers and may be omitted. |
| none | The same as `'p'`. |

## Chrono Format Specifications¶

Format specifications for chrono types have the following syntax:

```
chrono_format_spec ::=  [[fill]align][width]["." precision][chrono_specs]
chrono_specs       ::=  [chrono_specs] conversion_spec | chrono_specs literal_char
conversion_spec    ::=  "%" [modifier] chrono_type
literal_char       ::=  <a character other than '{', '}' or '%'>
modifier           ::=  "E" | "O"
chrono_type        ::=  "a" | "A" | "b" | "B" | "c" | "C" | "d" | "D" | "e" | "F" |
                        "g" | "G" | "h" | "H" | "I" | "j" | "m" | "M" | "n" | "p" |
                        "q" | "Q" | "r" | "R" | "S" | "t" | "T" | "u" | "U" | "V" |
                        "w" | "W" | "x" | "X" | "y" | "Y" | "z" | "Z" | "%"
```

Literal chars are copied unchanged to the output. Precision is valid only for
`std::chrono::duration` types with a floating-point representation type.

The available presentation types (*chrono\_type*) for chrono durations and time
points are:

| Type | Meaning |
| --- | --- |
| `'H'` | The hour (24-hour clock) as a decimal number. If the result is a single digit, it is prefixed with 0. The modified command `%OH` produces the locale’s alternative representation. |
| `'M'` | The minute as a decimal number. If the result is a single digit, it is prefixed with 0. The modified command `%OM` produces the locale’s alternative representation. |
| `'S'` | Seconds as a decimal number. If the number of seconds is less than 10, the result is prefixed with 0. If the precision of the input cannot be exactly represented with seconds, then the format is a decimal floating-point number with a fixed format and a precision matching that of the precision of the input (or to a microseconds precision if the conversion to floating-point decimal seconds cannot be made within 18 fractional digits). The character for the decimal point is localized according to the locale. The modified command `%OS` produces the locale’s alternative representation. |

Specifiers that have a calendaric component such as `'d'` (the day of month)
are valid only for `std::tm` and not durations or time points.

`std::tm` uses the system’s strftime so refer to its
documentation for details on supported conversion specifiers.

## Format Examples¶

This section contains examples of the format syntax and comparison with
the printf formatting.

In most of the cases the syntax is similar to the printf formatting, with the
addition of the `{}` and with `:` used instead of `%`.
For example, `"%03.2f"` can be translated to `"{:03.2f}"`.

The new format syntax also supports new and different options, shown in the
following examples.

Accessing arguments by position:

```
fmt::format("{0}, {1}, {2}", 'a', 'b', 'c');
// Result: "a, b, c"
fmt::format("{}, {}, {}", 'a', 'b', 'c');
// Result: "a, b, c"
fmt::format("{2}, {1}, {0}", 'a', 'b', 'c');
// Result: "c, b, a"
fmt::format("{0}{1}{0}", "abra", "cad");  // arguments' indices can be repeated
// Result: "abracadabra"
```

Aligning the text and specifying a width:

```
fmt::format("{:<30}", "left aligned");
// Result: "left aligned                  "
fmt::format("{:>30}", "right aligned");
// Result: "                 right aligned"
fmt::format("{:^30}", "centered");
// Result: "           centered           "
fmt::format("{:*^30}", "centered");  // use '*' as a fill char
// Result: "***********centered***********"
```

Dynamic width:

```
fmt::format("{:<{}}", "left aligned", 30);
// Result: "left aligned                  "
```

Dynamic precision:

```
fmt::format("{:.{}f}", 3.14, 1);
// Result: "3.1"
```

Replacing `%+f`, `%-f`, and `% f` and specifying a sign:

```
fmt::format("{:+f}; {:+f}", 3.14, -3.14);  // show it always
// Result: "+3.140000; -3.140000"
fmt::format("{: f}; {: f}", 3.14, -3.14);  // show a space for positive numbers
// Result: " 3.140000; -3.140000"
fmt::format("{:-f}; {:-f}", 3.14, -3.14);  // show only the minus -- same as '{:f}; {:f}'
// Result: "3.140000; -3.140000"
```

Replacing `%x` and `%o` and converting the value to different bases:

```
fmt::format("int: {0:d};  hex: {0:x};  oct: {0:o}; bin: {0:b}", 42);
// Result: "int: 42;  hex: 2a;  oct: 52; bin: 101010"
// with 0x or 0 or 0b as prefix:
fmt::format("int: {0:d};  hex: {0:#x};  oct: {0:#o};  bin: {0:#b}", 42);
// Result: "int: 42;  hex: 0x2a;  oct: 052;  bin: 0b101010"
```

Padded hex byte with prefix and always prints both hex characters:

```
fmt::format("{:#04x}", 0);
// Result: "0x00"
```

Box drawing using Unicode fill:

```
fmt::print(
  "┌{0:─^{2}}┐\n"
  "│{1: ^{2}}│\n"
  "└{0:─^{2}}┘\n", "", "Hello, world!", 20);
```

prints:

```
┌────────────────────┐
│   Hello, world!    │
└────────────────────┘
```

Using type-specific formatting:

```
#include <fmt/chrono.h>

auto t = tm();
t.tm_year = 2010 - 1900;
t.tm_mon = 7;
t.tm_mday = 4;
t.tm_hour = 12;
t.tm_min = 15;
t.tm_sec = 58;
fmt::print("{:%Y-%m-%d %H:%M:%S}", t);
// Prints: 2010-08-04 12:15:58
```

Using the comma as a thousands separator:

```
#include <fmt/locale.h>

auto s = fmt::format(std::locale("en_US.UTF-8"), "{:L}", 1234567890);
// s == "1,234,567,890"
```

© Copyright 2012-present, Victor Zverovich.
Created using Sphinx 3.3.0.
